# The Evaluation of Small-Scale Field Maize Transpiration Rate from UAV Thermal Infrared Images Using Improved Three-Temperature Model

**DOI:** 10.3390/plants14142209

**Published:** 2025-07-17

**Authors:** Xiaofei Yang, Zhitao Zhang, Qi Xu, Ning Dong, Xuqian Bai, Yanfu Liu

**Affiliations:** 1 College of Water Resources and Architectural Engineering, Northwest A&F University, Yangling 712100, China; xiaofei-yang@nwafu.edu.cn (X.Y.); 0405@nwafu.edu.cn (N.D.); baixuqian0628@nwafu.edu.cn (X.B.);; 2 Key Laboratory of Agricultural Soil and Water Engineering in Arid and Semiarid Areas, Ministry of Education, Northwest A&F University, Yangling 712100, China; 3 Xinjiang Research Institute of Agriculture in Arid Areas, Urumqi 830091, China

**Keywords:** transpiration rate, three-temperature model, thermal infrared remote sensing, unmanned aerial vehicle, GHMRF model

## Abstract

Transpiration is the dominant process driving water loss in crops, significantly influencing their growth, development, and yield. Efficient monitoring of transpiration rate (Tr) is crucial for evaluating crop physiological status and optimizing water management strategies. The three-temperature (3T) model has potential for rapid estimation of transpiration rates, but its application to low-altitude remote sensing has not yet been further investigated. To evaluate the performance of 3T model based on land surface temperature (LST) and canopy temperature (T_C_) in estimating transpiration rate, this study utilized an unmanned aerial vehicle (UAV) equipped with a thermal infrared (TIR) camera to capture TIR images of summer maize during the nodulation-irrigation stage under four different moisture treatments, from which LST was extracted. The Gaussian Hidden Markov Random Field (GHMRF) model was applied to segment the TIR images, facilitating the extraction of T_C_. Finally, an improved 3T model incorporating fractional vegetation coverage (FVC) was proposed. The findings of the study demonstrate that: (1) The GHMRF model offers an effective approach for TIR image segmentation. The mechanism of thermal TIR segmentation implemented by the GHMRF model is explored. The results indicate that when the potential energy function parameter β value is 0.1, the optimal performance is provided. (2) The feasibility of utilizing UAV-based TIR remote sensing in conjunction with the 3T model for estimating Tr has been demonstrated, showing a significant correlation between the measured and the estimated transpiration rate (T_r_-3T_C_), derived from T_C_ data obtained through the segmentation and processing of TIR imagery. The correlation coefficients (r) were 0.946 in 2022 and 0.872 in 2023. (3) The improved 3T model has demonstrated its ability to enhance the estimation accuracy of crop Tr rapidly and effectively, exhibiting a robust correlation with T_r_-3T_C_. The correlation coefficients for the two observed years are 0.991 and 0.989, respectively, while the model maintains low RMSE of 0.756 mmol H_2_O m^−2^ s^−1^ and 0.555 mmol H_2_O m^−2^ s^−1^ for the respective years, indicating strong interannual stability.

## 1. Introduction

Water scarcity is a major challenge hindering the sustainable development of agriculture. Enhancing water use efficiency in agricultural production while simultaneously ensuring the balanced development of food security and the ecological environment is a critical issue requiring urgent attention [1,2]. Crop transpiration is a vital physiological process in agricultural systems, acting as the primary mechanism for water loss in crops and significantly influencing crop growth, development, and yield formation [3,4]. Despite its importance, several challenges persist in the monitoring of transpiration rate (Tr). These include low spatial resolution, high equipment and operational costs, and significant sensitivity to meteorological conditions. Therefore, developing methods for estimating crop transpiration rates in a cost-effective, rapid, and accurate manner is essential for monitoring crop physiological status and managing agricultural water resources.

Existing methods for quantifying crop transpiration include water balance, plant physiological, and micrometeorological approaches. The water balance and plant physiological methods rely on specialized equipment, such as weighing lysimeters, sap flow meters, and leaf chamber gas exchange systems, to measure Tr at the leaf or canopy level [5,6,7]. While these methods provide high-precision data, they suffer from several limitations. These include complex operation, high costs, and low spatial and temporal resolution, which restrict their applicability in large-scale agricultural settings. Micrometeorological methods, on the other hand, typically use meteorological and surface flux data to estimate Tr. Common techniques, such as the energy balance and aerodynamic methods [8,9], offer continuous measurements with high temporal resolution. However, these methods require high-quality input parameters, which can be difficult to obtain—particularly parameters like aerodynamic resistance, thus limiting their large-scale implementation at large scales. Moreover, these methods face spatial limitations spatial limitations. For example, the water balance method provides an average value over the test area and cannot account for variations among individual plants. These challenges hinder the ability to monitor crop transpiration with high precision, efficiency, and cost-effectiveness.

Remote sensing technology provides novel approaches for monitoring crop Tr, with thermal infrared (TIR) remote sensing showing particular promise [10,11,12,13]. Since its development, satellite-borne TIR sensors, in combination with evapotranspiration modeling, have been used to estimate surface evapotranspiration. However, their applicability at the agricultural scale remains limited [14,15,16]. In contrast, ground-based TIR imaging systems offer higher temporal resolution and improved precision. For example, Tian, et al. [17] employed handheld TIR sensors to estimate Tr for 16 vegetation species in the Gobi Desert, showing a strong correlation with measured values, although spatial limitations were noted. Recently, the rapid advancement of TIR remote sensing technology using unmanned aerial vehicles (UAVs) has enabled the efficient monitoring of crop transpiration across large agricultural areas. This technology provides several advantages, including high spatial resolution, flexibility, maneuverability, and relatively low cost [18,19].

The use of UAVs equipped with TIR sensors enables the direct measurement of land surface temperature (LST) in agricultural areas [20], which is essential for estimating crop Tr. However, the direct application of LST in transpiration estimation may be prone to errors due to incomplete canopy closure. TIR imagery capture both canopy and soil components [21], and the soil temperature can influence the accuracy of Tr estimates. Currently, a primary focus of TIR remote sensing research is the extraction of crop T_C_ from farmland images. This process typically involves segmenting and processing TIR images to isolate canopy pixels. Two main approaches are used: direct and indirect methods. Direct method, such as thresholding and edge detection [22,23,24]—are simple and convenient but often yield lower segmentation accuracy. Indirect method involve aligning visible images with TIR images and applying a visible-band image mask to the TIR data [25,26]; although more accurate, these methods face challenges in achieving precise pixel-level alignment. Zhang, et al. [27] proposed the Gaussian Hidden Markov Random Model (GHMRF), initially developed for brain magnetic resonance imaging (MRI) and more recently adapted for radar remote sensing image processing, achieving improved segmentation results [28]. However, the potential applications and optimal parameter selection of GHMRF in TIR remote sensing remain underexplored.

Currently, the primary method for monitoring crop Tr using UAV-based TIR sensing involves integrating energy balance models, such as the Two-Source Energy Balance (TSEB) model, the Penman model, and the three-temperature (3T) model. Both the TSEB and Penman models face limitations due to their reliance on complex parameters. In contrast, the 3T model, proposed by Qiu, et al. [29], incorporates reference leaf temperature to address the issue of limited parameter availability.

The model requires only four key input variables: net solar radiation, T_C_, reference leaf temperature, and atmospheric temperature. This simplification enhances its suitability for application in TIR remote sensing. The 3T model enable large-scale, non-invasive monitoring of crop Tr via UAV-based TIR measurement of canopy temperature, supplemented by data from ground-based meteorological instruments [30]. However, the temperature obtained from UAV-based TIR remote sensing is LST, not T_C_. Therefore, it is important to examine whether LST can be directly used as an input in the 3T model for estimating Tr. If the use of LST results in inaccurate estimates, an alternative approach involve incorporating crop phenotypic parameters into the 3T model to improve its applicability for estimating Tr via UAV-based TIR remote sensing.

The principal objectives of this study were to: (1) evaluate the effectiveness of the GHMRF model for TIR image segmentation; (2) assess the potential of UAV-based TIR remote sensing combined with the 3T model for estimating Tr; and (3) enhance the 3T model by incorporating crop phenotypic parameters to improving its applicability for estimating crop Tr using UAV-based TIR remote sensing.

## 2. Materials and Methods

### 2.1. Overview of the Experimental Area

**Figure 1:**
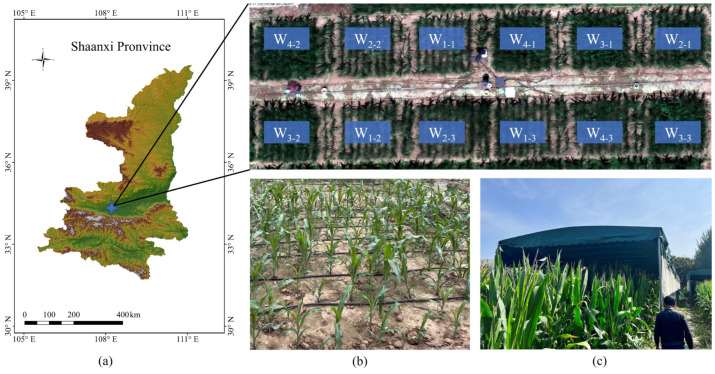
Layout of the experimental area (**a**) Study site: maize experiment in 2023; (**b**) Drip irrigation system; (**c**) Mobile awning, W_1_, W_2_, W_3_, W_4_ denote irrigation gradients with upper limit of the irrigation of 50%, 65%, 80%, and 95%, respectively, and W_1-1_ denotes the first plot in the W_1_ irrigation treatment, and the others as well.

The experiment was conducted at the Institute of Water-saving Agriculture in Arid Zones of China, Northwest A&F University (Figure 1a). The region experiences a continental warm-temperate monsoon climate, characterized by warm, windy springs and a hot, rainy summers. The average annual temperature is 12.9 °C, and the annual precipitation is approximately 600 mm. The soil in the experimental area is medium loam, with an average field capacity of 21.5% and a wilting coefficient of 6.7% (both in mass water content).

The experiment consisted of 12 plots, each measuring 4 m × 4 m, subjected to four different moisture treatments. The irrigation upper limit for these treatments were set at 50% (W1), 65% (W2), 80% (W3), and 95% (W4) of the field capacity. This design ensured consistent and controlled water stress levels across treatments, facilitating analysis of maize physiological and thermal responses under varying irrigation regimes. Three replicates were established for each treatment (Figure 1a). Protective rows, 1 m wide and planted with two rows of maize, were established between adjacent plots in the east-west direction to minimize water interpenetration (Figure 1a). To eliminate the influence of natural precipitation, a collapsible mobile rain shelter was constructed over the experimental area (Figure 1b). Each plot was equipped with a drip irrigation system and a water meter for precise irrigation control (Figure 1c).

A two-year field experiment was conducted. In 2022, maize was sown on 21 June and harvested on 30 September, with a growing period of 101 days. In 2023, maize was sown on 16 June and harvested on 8 October, spanning 114 days. The seeds were planted in rows with 50 cm spacing between rows and 25 cm between plants within each row.

### 2.2. Data Collection

To ensure data reliability, the experiment was conducted during periods of optimal sunlight and minimal cloud cover. In 2022, data collection occurred between 29 July and 25 August, during which nine sets of UAV-based TIR data and six sets of ground-based transpiration data were collected. In 2023, data collection took place between 3 August and 1 September, yielding 12 sets of UAV-based TIR data and three sets of ground-based transpiration data collected.

#### 2.2.1. Thermal Infrared Data

**Figure 2:**
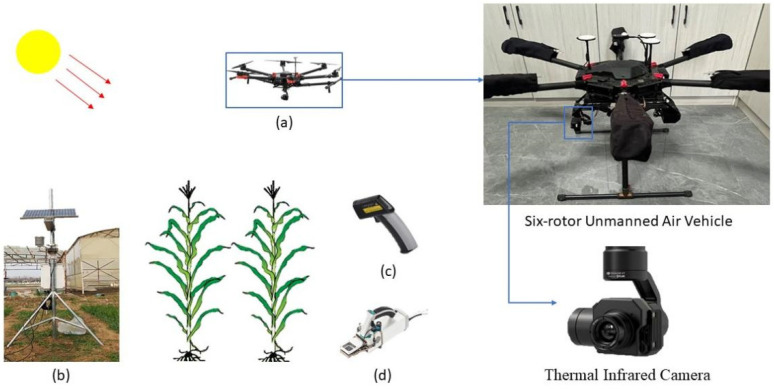
Experimental instruments (**a**) UAV thermal infrared system; (**b**) weather station; (**c**) handheld thermal infrared thermometer; (**d**) LI-6800 portable photosynthesis meter.

The experiment utilized a hexacopter UAV DJI Matrice 600 Pro (DJI Technology Co., Shenzhen, China) equipped with a TIR camera ZENMUSE XT (DJI Technology Co., Shenzhen, China) to collect TIR data (Figure 2a). The UAV gimbal pitch angle was set to −90°, with both the forward and side overlap rates configured at 80%. The TIR camera operates within a wavelength range of 7.5–13.5 μm, with a pixel resolution of 640 × 512, a horizontal field of view of 32°, a vertical field of view of 26°, a lens focal length of 19 mm, and a thermal sensitivity of 0.05 °C. TIR data were collected during UAV flights at 13:00 on the data collection day, with a flight altitude of 20 m. At this altitude, the image resolution was approximately 1.8 cm. The time of 13:00 was selected because it typically corresponds to peak solar radiation and maximal canopy temperature, which enhances the thermal contrast between the crop and soil.

#### 2.2.2. Meteorological Data

A small meteorological station was installed in the experimental area to collect meteorological data (Figure 2b). Measurements were collected at two-minute intervals. The primary meteorological variables recorded included atmospheric temperature (Ta, °C), solar radiation (Rn, W/m^2^), relative humidity (RH, %), and wind speed (WS, m/s). The types of sensors used in the meteorological station and their respective measurement accuracies are listed in Table 1.

#### 2.2.3. Ground Temperature Data

While the UAV collected TIR data, ground temperature measurements were taken using a handheld TIR thermometer (RayTek ST60+, RayTek Inc., Santa Cruz, CA, USA) (Figure 2c), which operates within a spectral response range of 8–14 μm and has an emissivity setting of 0.97. Ground temperature measurements were recorded for T_C_, water column temperature, and reference leaf temperature. The ground-measured T_C_ was used to evaluate the T_C_ extracted by TIR remote sensing. The water column temperature was used to calibrate the TIR images, while the reference leaf temperature was employed in calculating the Tr.

#### 2.2.4. Crop Physiological Indicator Data

The physiological indices of summer maize were measured using a portable photosynthesis system (LI-6800, LI-COR Inc., Lincoln, NE, USA) (Figure 2d). The primary indices included net photosynthetic rate (*A*, μmol m^−2^ s^−1^), stomatal conductance (*G_s_*, mol m^−2^ s^−1^), and transpiration rate (*T_r_*, mol m^−2^ s^−1^). Approximately 30 min before and after each UAV flight, three maize plants were selected diagonally within the experimental plots. Three leaves from each plant were used to determine the Tr using the LI-6800. The average transpiration rate (T_r_-LI) across all measurement points within the plots was then calculated.

### 2.3. Thermal Infrared Data Processing

#### 2.3.1. Data Pre-Processing

The TIR images collected by the UAV were imported into FLIR Tools software, where the image greyscale values were converted into temperature data. The measured water temperature was then used to calibrate the TIR image temperatures. A linear regression was performed to establish the relationship between the greyscale values and the calibrated temperatures, which was subsequently used to calculate the temperature of the canopy pixels.

#### 2.3.2. Segmentation of Image Features

**Figure 3:**
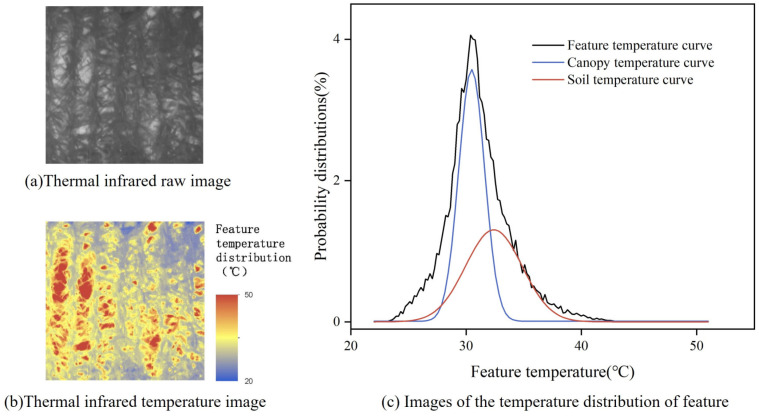
Temperature distribution of thermal infrared images. (**a**,**b**) Thermal infrared images and (**c**) temperature distribution of feature.

In TIR remote sensing of farmland, the target scene typically consists of two components: the crop canopy and the soil. Some researchers have suggested that the temperature distribution of these components follows a Gaussian Mixture Model (GMM) [31], which is also applicable to the canopy and soil temperatures in this experiment. The temperature distribution can be described by the following equation:

(1) $$GMM=A\times N\left(\mu_{c},\sigma_{c}^{2}\right)+B\times N\left(\mu_{s},\sigma_{s}^{2}\right)$$

where A and B represent the weights of the temperature distributions for the canopy and soil, respectively. *σ* and *μ* denote the mean and standard deviation of a Gaussian distribution, respectively. The subscripts c and s refer to the canopy and soil components. $N\left(\mu ,\sigma^{2}\right)$ denotes the Gaussian distribution function. As shown in Figure 3b, the temperature distribution consists of two components: the overall temperature distribution curve, represented by the black curve in Figure 3c, and the low temperature canopy component, represented by the blue curve in Figure 3c. The high-temperature soil component is indicated by the red curve in Figure 3c.

In this study, the Gaussian Hidden Markov Random Field (GHMRF) model [27] was used for TIR image segmentation. This model integrates the spatial dependence of the Hidden Markov model (HMM), and the spatial consistency of the Markov Random Field (MRF), thereby enhancing robustness and reducing sensitivity to erroneous responses. The GHMRF model considers the spatial relationship between each pixel point and its neighborhood, which is formulated as:

(2) $$p\left(X_{i}|x_{Ni};\theta \right)=\sum_{l\in L}g\left(X_{i};\theta_{l}\right)q\left(l|x_{Ni}\right)$$

where $X_{i}$ is the value of pixel i, $x_{Ni}$ represents the set of neighboring pixels of i, θ denotes the model parameters (mean *μ*, variance *σ*^2^), *L* is the number of segmentations labels, g is the Gaussian distribution function and q is the probability density function.

The segmentation image was updated using the Maximum A Posteriori (MAP) algorithm [32], formulated as:

(3) $$\hat{x}=\underset{x\in \chi }{\mathrm{argmin}}\left\{U\left(X_{i}|x\right)+U\left(X\right)\right\}$$

where $\hat{x}$ is the resulting segmentation of the image, χ is the set of all possible label configurations. $U\left(X_{i}|x\right)$ denotes the likelihood energy function. U(X) denotes the prior energy function, the function is defined as:

(4) $$U\left(X\right)=\beta \cdot \sum_{(i,j)\in \chi }II[x_{i}\neq x_{j}]$$

where $x_{i}$ and $x_{j}$ represent the labels of neighboring pixels i and j, and β is a non-negative scalar parameter that controls the degree of spatial regularization.

An iterative optimization process was performed using the Expectation-Maximization (EM) algorithm [33]. In the E-step, the MAP estimation was used to update the bias field and image segmentation labels. In the M-step, maximum likelihood (ML) estimation of the model parameter θ was performed, using the estimated bias field and segmentation results from the E-step. The calculations are expressed as follows:

E-step

(5) $$MAP:B^{\left(t\right)}=arg\;\underset{B}{\max}\;p\left(B|{X,x}^{\left(t-1\right)},\theta^{\left(t\right)}\right)$$

(6) $$MAP:x^{\left(t\right)}=arg\;\underset{x\in \chi }{\min}\;U\left(x|{X,B}^{\left(t\right)},\theta^{\left(t\right)}\right)$$

M-step

(7) $$ML:\theta^{\left(t+1\right)}=arg\;\underset{\theta }{\max}\;P\left(X|{\theta ,x}^{\left(t\right)},B^{\left(t\right)}\right)$$

#### 2.3.3. Fractional Vegetation Coverage

In this study, since the resolution of the TIR images was lower than the width of a maize leaf, it was assumed that the image pixels primarily represent pure pixels [18]. Based on the definition of Fractional Vegetation Coverage (FVC) for crops, the calculation formula is as follows:

(8) $$FVC=\frac{S_{C}}{S_{a}}$$

where $S_{C}$ is the number of canopy pixels, and $S_{a}$ is the total number of pixels in the image.

### 2.4. Crop Transpiration Rate

The three-temperature (3T) model, proposed by Qiu, Momii and Yano [29], is capable of estimating Tr with reasonable accuracy and requires fewer input parameters, making it suitable for regions where meteorological data is lacking. The 3T model is based on the surface energy balance equation, which is expressed as:

(9) $$\lambda ET=R_{n}-G-H$$

where $R_{n}$ is the net radiation flux (W·m^−2^), G is the soil heat flux (W·m^−2^), H is the sensible heat flux (W·m^−2^), and λET represents the latent heat flux used for soil evaporation (E) and plant transpiration (T) (W·m^−2^), where λ is the latent heat of vaporization taken as 2.45 × 106 W·m^−2^·mm^−1^.

In this study, summer maize leaves without transpiration were modeled by introducing a reference leaf, based on which the aerodynamic resistance $r_{a}$ was calculated. The canopy sensible heat flux $H_{c}$ and aerodynamic resistance $r_{a}$ were calculated as follows:

(10) $$H_{c}=\frac{\rho C_{p}\left(T_{c}-T_{a}\right)}{r_{a}}$$

(11) $$r_{a}=\frac{\rho C_{p}\left(T_{p}-T_{a}\right)}{R_{n,p}}$$

where ρ is the air density (kg·m^−3^), $C_{p}$ is the specific heat capacity at constant pressure (MJ·kg^−1^·K^−1^), $T_{c}$, $T_{a}$ and $T_{p}$ represent the canopy, atmospheric and reference leaf temperatures (K), respectively, and $R_{n,p}$ is the net radiation flux absorbed by the reference leaf (W·m^−2^).In this experiment, only transpiration of summer maize was monitored on a daily scale, so evaporation (E) and soil heat flux (G) were considered negligible. Substituting of Equations (10) and (11) into energy balance Equation (8) yields:

(12) $$\lambda T=R_{n,c}-R_{n,p}\left(\frac{T_{c}-T_{a}}{T_{p}-T_{a}}\right)$$

Since the experimental area was small and both the reference leaf and the maize canopy were located within the same area—with observation scales are at the centimeter level [34], it was assumed that they received the same net radiation flux. Therefore, Equation (9) can be simplified to:

(13) $$\lambda T=R_{n,p}\left(1-\frac{T_{c}-T_{a}}{T_{p}-T_{a}}\right)$$

The Tr calculated using Equation (12) is initially expressed in units of mm/h. To conform with standard conventions in plant physiology and remote sensing, this value is converted to mmol H_2_O m^−2^ s^−1^ using the following formula:

(14) $$1\;mmol\;H_{2}O\;m^{-2}s^{-1}=15.43\;mm\;H_{2}O/h$$

### 2.5. Accuracy Evaluation

#### 2.5.1. Evaluation of Image Segmentation Accuracy

Segmentation accuracy in this study was evaluated using Overall Accuracy (OA), Kappa coefficient, User’s Accuracy (UA), and Producer’s Accuracy (PA).

OA represents the proportion of correctly labeled pixels (either canopy or soil) to the total number of pixels in the segmented image, and is calculated as:

(15) $$OA=\frac{TP+TN}{TP+TN+FP+FN}$$

where TP (true positives) refers to the number of canopy pixels correctly segmented as canopy; TN (true negatives) refers to the number of soil pixels correctly segmented as soil; FP (false positives) is the number of soil pixels incorrectly segmented as canopy; and FN (false negatives) is the number of canopy pixels incorrectly segmented as soil.

KC quantifies the agreement between the segmentation result and ground truth, corrected for random chance. It is calculated as follows:

(16) $$KC=\frac{OA-PE}{1-PE}$$

where PE the expected accuracy by random chance, is calculated as:

(17) $$PE=\frac{\left(TP+FP\right)\left(TP+FN\right)+\left(TN+FP\right)\left(TN+FN\right)}{(TP+TN+FP+FN{)}^{2}}$$

UA indicates the reliability of the segmented canopy region, defined as the proportion of pixels segmented as canopy that are indeed canopy:

(18) $$UA=\frac{TP}{TP+FP}$$

PA indicates how well the true canopy pixels were detected by the segmentation, calculated as:

(19) $$PA=\frac{TP}{TP+FN}$$

#### 2.5.2. Evaluation of Model Accuracy

In this study, model performance was evaluated using the correlation coefficient (r) and the root-mean-square error (RMSE). The correlation coefficient measures the strength of the linear relationship between two variables, with values closer to 1 indicating stronger correlation. The RMSE quantifies the error between estimated and observed values, with smaller values indicating higher accuracy.

The formulas used to calculate *r* and RMSE are as follows:

(20) $$r=\frac{\sum \left(x_{i}-\bar{x}\right)\left(y_{i}-\bar{y}\right)}{\sqrt{\sum {\left(x_{i}-\bar{x}\right)}^{2}\cdot \sum {\left(y_{i}-\bar{y}\right)}^{2}}}$$

(21) $$RMSE=\sqrt{\frac{\sum_{i=1}^{n}{\left({\hat{y}}_{i}-y_{i}\right)}^{2}}{n}}$$

## 3. Results and Analysis

### 3.1. Results of the Field Measurements

#### 3.1.1. Basic Meteorological Characteristics

**Figure 4:**
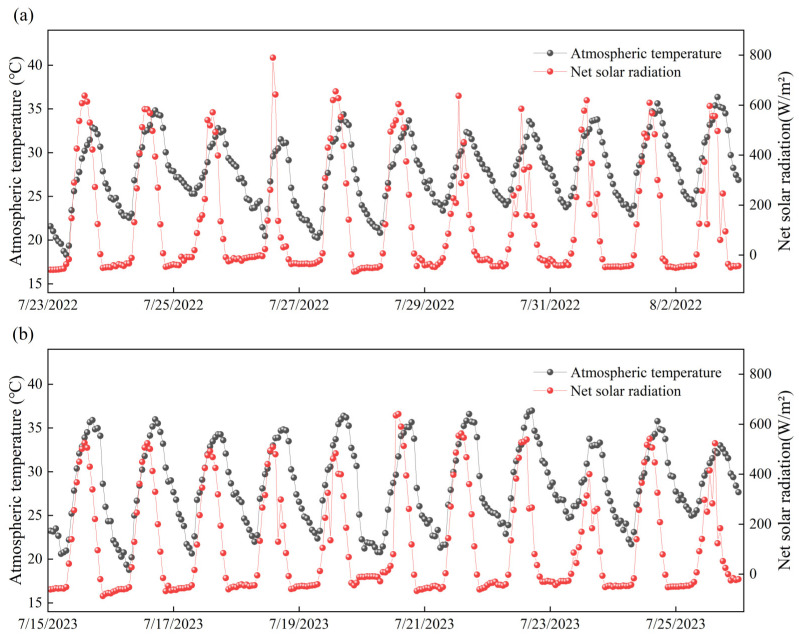
Daily change of partial temperature and net solar radiation measured during the (**a**) 2022 and (**b**) 2023 experiment.

During the two-year experimental period, a distinct diurnal pattern in atmospheric temperature was observed. As shown in Figure 4, the atmospheric temperature reached its minimum around 6:00 a.m., ranging between 18 °C and 23 °C. With the increase in solar radiation, the temperature rose steadily, peaking around 3:00 p.m. at 33–37 °C. It then declined, the temperature declined, returning to the daily minimum by the following morning.

The daily pattern of net solar radiation closely mirrored that of the atmospheric temperature, though the timing of the peak differed slightly. After sunrise, net radiation increased rapidly, reaching a daily maximum of 400–800 W/m^2^ around 1:00 p.m. It then decreased, reaching a minimum of −100 to 0 W/m^2^ after sunset. During the nighttime period—from sunset to sunrise—fluctuations in net radiation were primarily driven by longwave radiation emitted from both the atmosphere and the land surface. These variations exhibited a narrower range and can be considered the daily minimum.

#### 3.1.2. Measured Canopy Temperature and Transpiration Rate

**Figure 5:**
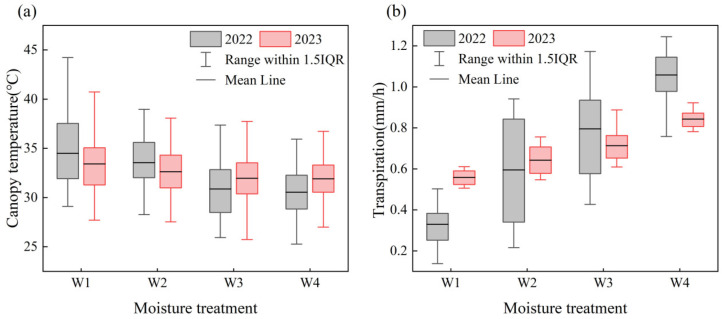
Measured (**a**) canopy temperature and (**b**) transpiration rate under different moisture treatments.

The statistical analysis of the measured T_C_ and Tr under different moisture treatments in 2022 and 2023 is presented in Figure 5. The results revealed significant differences in T_C_ across the moisture treatments in both years (Figure 5a). Specifically, T_C_ decreased with increasing soil moisture, indicating that higher soil moisture effectively lowers the T_C_ of summer maize. Similarly, Tr showed substantial variation across the moisture treatments (Figure 5b). In contrast to the trend observed in T_C_, Tr increased markedly with higher soil moisture, suggesting that elevated moisture enhances the Tr of summer maize.

Overall, both T_C_ and Tr were strongly correlated with soil moisture levels. These findings suggest that increasing soil moisture not only reduces T_C_ but also improves the Tr of summer maize, thereby improving its thermal regulation and physiological activity.

### 3.2. Evaluation of Segmentation Accuracy of TIR Images

The GHMRF model employs a potential energy function to quantify the spatial relationships between each image element and its neighboring domain. The parameter *β* in this function plays a critical role in controlling the degree of similarity enforced between adjacent pixels, thereby promoting spatial consistency in segmentation results. In this study, *β* values were varied from 0.001 and 1.0 to assess their influence on the segmentation results of the GHMRF model. The goal was to identify the optimal *β* value for segmenting TIR images of agricultural fields. The results are summarized in Table 2.

As shown in Table 2, the segmentation accuracy of the GHMRF model varies with changes in the *β* value. At *β* = 0.1, the model achieves the highest segmentation accuracy, with an OA of 94.80% and a KC of 88.51%. Across the *β* range of [0.001, 1.0], both OA and PA increase as *β* increases, peaking at *β* = 0.1 before stabilizing or declining. Meanwhile, UA rises steadily with increasing *β* until it peaks at *β* = 0.8, after which it stabilizes. This trend may be explained by the fact that, as *β* increases, the edges of object classes become smoother, causing a gradual reduction in the number of pixels classified as canopy. While this enhances user accuracy, it leads to inconsistencies with the trends observed in OA, KC, and PA, which are more sensitive to canopy omission errors.

### 3.3. Spatial and Temporal Distribution of LST and T_C_

**Figure 6:**
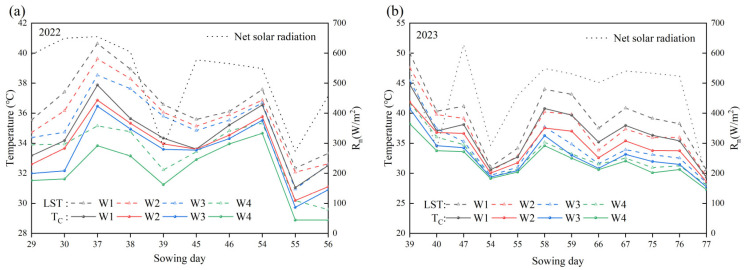
Temporal change of land surface temperature and canopy temperature (**a**) data from 2022; (**b**) data from 2023.

In this study, UAV-based TIR remote sensing was employed to obtain the LST of the experimental plots. The GHMRF model was utilized to segment and process the TIR images, thereby generating a canopy image for each experimental plot. The corresponding T_C_ was then extracted. The statistical analyses of surface temperature, canopy temperature, and solar radiation at the same moment in time, as illustrated in Figure 6, demonstrate that the trends of LST and T_C_ exhibit a robust correlation with the net solar radiation. This is because the primary source of surface heating is incoming shortwave solar radiation.

The effects of different moisture treatments on LST and T_C_ were further investigated. The results demonstrate that moisture treatments exerted a significant influence on LST and T_C_. As illustrated in Figure 6, LST and T_C_ were consistently highest under the W1 treatment, followed by the W_2_ and W_3_ treatments in that order. In contrast, both remained at a lower level in the W_4_ treatment. This phenomenon can be attributed to the following factors. In the W_4_ irrigation treatment, soil moisture was sufficient, soil specific heat capacity was higher, the heat transfer rate was faster, and summer maize transpiration was enhanced, resulting in lower LST and T_C_. Conversely, In the W1 treatment, there was a deficiency in soil moisture, a reduction in soil specific heat capacity, a slowing of the heat transfer rate, and a weakening of summer maize transpiration, which collectively resulted in higher LST and T_C_.

Furthermore, LST values were consistently higher than T_C_ under either moisture treatment, as illustrated in Figure 6. This is due to the fact that when the canopy is not fully closed, the canopy and the soil are exposed to solar radiation at the same time. The summer maize canopy has a higher albedo relative to the soil, resulting in less heat absorption, thereby producing a soil temperature higher than the T_C_. The T_C_ is significantly lower than the soil temperature due to the strong transpiration cooling effect of the summer maize canopy. The LST is the combined result of the T_C_ and soil temperature. Consequently, there is a phenomenon whereby the T_C_ is lower than the LST.

**Figure 7:**
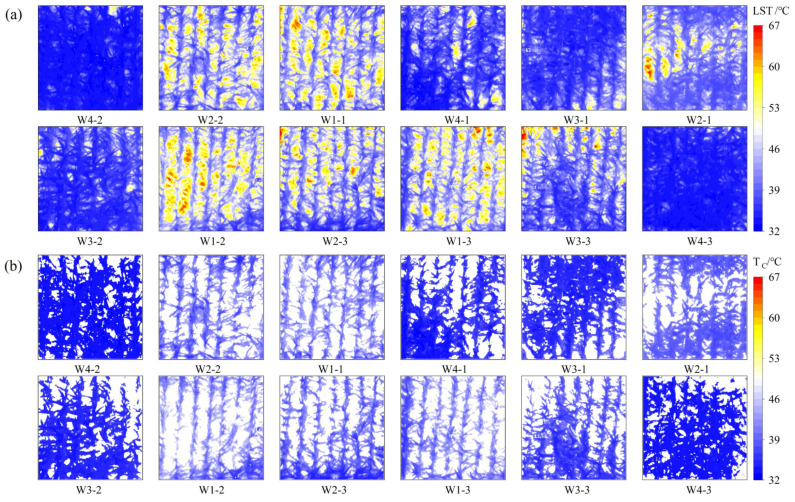
Spatial changes of land surface temperature (**a**) and canopy temperature (**b**), using August 14, 2023 data as an example, W_1_, W_2_, W_3_, W_4_ denote irrigation gradients with irrigation ceilings of 50%, 65%, 80%, and 95%, respectively, and W_1-1_ denotes the first plot in the W_1_ irrigation gradient, and the others as well.

Concurrently, this study analyzed the spatial distribution of LST and T_C_. The results are presented in Figure 7, which demonstrates that both LST and T_C_ of plots subjected to different moisture treatments exhibited significant differences in spatial distribution. From Figure 7a, it can be observed that with the alteration of the moisture treatment, the temperature extremes within the experimental area also changed. For instance, the maximum temperatures of the W1 treatments reached above 65 °C, whereas the maximum temperature of the W4 treatments was approximately 53 °C, with a notable difference of 12 °C. Furthermore, the temperature maxima in the LST were observed in the soil region. In the W4 treatment, an increase in soil moisture resulted in a corresponding increase in the soil heat transfer rate. This treatment facilitated the growth of the summer maize, which exhibited high canopy cover and the ability to intercept a greater quantity of solar radiation. This, in turn, led to relatively low soil temperatures. The opposite was true in the W1 treatment, where the summer maize was subjected to severe water stress with curled leaves, reduced canopy cover, increased solar radiation to the soil, and low soil moisture that slowed down the rate of heat transfer and sustained high soil accumulation temperatures.

The summer maize T_C_ exhibited a distinct pattern of low temperatures in the center and high temperatures at the edges, influenced by the summer maize phenotypic traits, such as leaf inclination and chlorophyll content. This phenomenon was particularly evident in the W1 and W2 treatment plots (Figure 7b). TIR remote sensing provided a clear illustration of this discrepancy at the centimeter scale. In general, both LST and T_C_ exhibited notable spatial heterogeneity across different treatments. The observed temperature variation patterns were consistent with water stress conditions, with the T_C_ values increasing correspondingly with the degree of water stress.

### 3.4. Estimation of Transpiration Rate by the 3T Model Based on Thermal Infrared Remote Sensing from UAV

**Figure 8:**
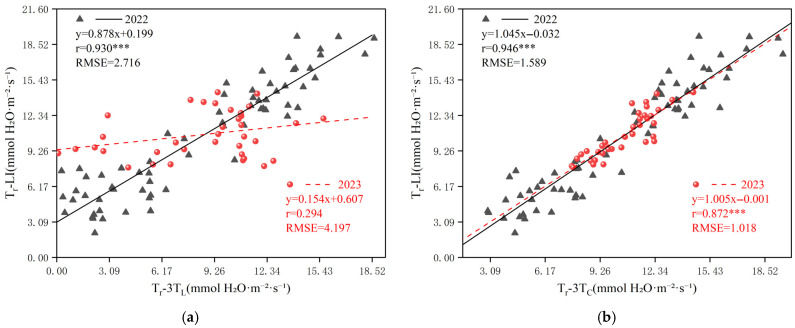
Relationship between measured and estimated transpiration rates (**a**) is the estimated transpiration rate using LST for 2022 (n = 72) and 2023 (n = 36) data, ((**b**) is the estimated transpiration rate using T_C_ for 2022(n = 72) and 2023 (n = 36) data). Note: *** indicates statistical significance at *p* < 0.001.

In this study, the two temperatures obtained by UAV-based TIR, LST and T_C_, were used as input parameters of the 3T model to derive the 3T model based on LST (3T_L_ model) and the 3T model based on T_C_ (3T_C_ model), respectively. To further evaluate the effectiveness of the 3T_L_ and 3T_C_ models in estimating T_r_, correlation analyses were conducted between T_r_-3T_L_, T_r_-3T_C_, and the actual measured transpiration rate (T_r_-LI). The outcomes of this analysis are illustrated in Figure 8.

The T_r_-3T_L_ showed substantial interannual variability, with a correlation coefficient (r) of 0.930 in 2022 and only 0.294 in 2023 (Figure 8a), and a root mean square error (RMSE) exceeding 2.50 mmol H_2_O m^−2^ s^−1^ in both years. In contrast, the T_r_-3T_C_ exhibited strong and consistent correlations across year, with r values of 0.946 in 2022 and 0.872 in 2023 (Figure 8b), and RMSE values significantly lower than 1.6 mmol H_2_O m^−2^ s^−1^. These results revealed a significant linear relationship between T_r_-3T_C_ and T_r_-LI, indicating a high accuracy and low error. These findings suggest that the 3T_C_ model exhibits enhanced accuracy and stability on an annual scale. Overall, a comparative analysis between the 3T_C_ and 3T_L_ models revealed that the 3T_C_ model yields significantly better estimation outcomes. This highlights the importance of precise canopy temperature extraction—enabled by thermal infrared image segmentation—for monitoring summer maize Tr using UAV-based TIR remote sensing.

**Figure 9:**
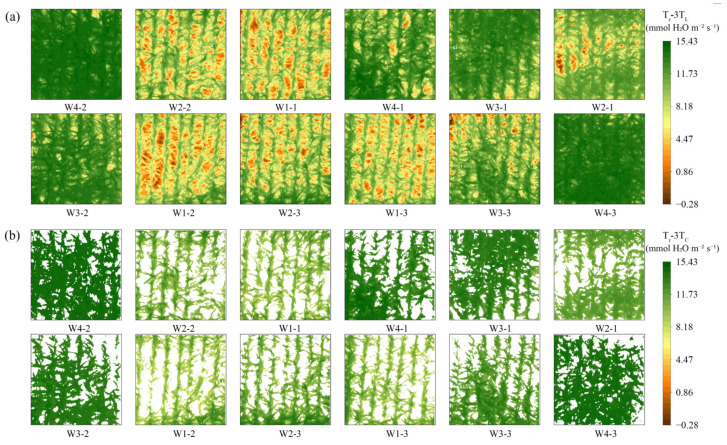
Spatial changes of T_r_-3T_L_ (**a**) and T_r_-3T_C_ (**b**), using 14 August 2023 data as an example, W_1_, W_2_, W_3_, W_4_ denote irrigation gradients with irrigation ceilings of 50%, 65%, 80%, and 95%, respectively, and W_1-1_ denotes the first plot in the W_1_ irrigation gradient, and the others as well.

Furthermore, this study analyzed the spatial distribution of crop Tr estimated by different models under varying moisture treatments (Figure 9) and conducted a descriptive statistical analysis(Table 3). It was observed that the T_r_ exhibited significant spatial variation and non-uniform distribution across the study area. Moreover, a positive correlation was observed between T_r_ and moisture treatment, with higher irrigation leading to increased T_r_s. As an illustration, the T_r_-3T_C_ values for the W1 treatment exhibited a range of 7.0–14.0 mmol H_2_O m^−2^ s^−1^, with a mean value of approximately 9.72 mmol H_2_O m^−2^ s^−1^. In comparison, the other moisture treatments demonstrated mean values that ranged from 10.0–15.5 mmol H_2_O m^−2^ s^−1^. In the same moisture treatment, the T_r_ demonstrated a decreasing pattern from the center of the summer maize to the edge, contrary to the spatial trend of T_C_. This phenomenon can be attributed to several factors, including the larger leaf inclination angle of the leaves at the center of the summer maize, lower solar radiation per unit leaf area, and weakened high-temperature dormancy of leaf stomata. These factors collectively result in the T_r_ remaining at a higher level. These differences indicate the spatial heterogeneity of summer maize canopy T_r_. UAV-based TIR remote sensing technology is able to capture such differences at the centimeter scale, providing strong support for high-precision monitoring of T_r_.

As illustrated in Table 2, there is a notable discrepancy between the descriptive statistics values of T_r_-3T_L_ and T_r_-3T_C_ within the same experimental map. This is particularly evident in the minimum value, where the difference between the two values reaches 12.04 mmol H_2_O m^−2^ s^−1^. As shown in Figure 9a, the low T_r_-3T_L_ values are mainly located in soil region. Since the statistical analysis includes both canopy and soil areas, this leads to a significant difference in the minimum values between T_r_-3T_L_ and T_r_-3T_C_, and in some cases, T_r_-3T_L_ values even fall below zero. In contrast, T_r_-3T_C_ (Figure 9b) excludes the soil background, and its statistics represent only the canopy area. This indicates that while the 3T model is applicable for estimating the Tr of the summer maize canopy, it is not suitable for use over bare soil surfaces. Including soil pixels in the statistical range of T_r_-3T_L_ causes the observed discrepancies. Furthermore, in thermal infrared images with lower canopy coverage, a larger proportion of soil pixels leads to a greater influence on the T_r_-3T_L_ statistical outcomes.

### 3.5. Improved 3T Model Based on Fractional Vegetation Coverage (FVC)

The findings of the aforementioned studies indicate that the estimation of summer maize Tr via UAV-based TIR remote sensing in conjunction with the 3T model is a feasible approach. To enhance the estimation accuracy, TIR images must be segmented and processed in order to derive summer maize T_C_. Nevertheless, the processing of TIR images is constrained by the limitations associated with low-accuracy images acquired over extensive areas and at elevated flight altitudes.

To address these issues, this study enhances the 3T_L_ model, which has been demonstrated to be a rapid estimator of summer maize Tr, albeit with considerable error in its estimates, by introducing the concept of Fractional Vegetation Cover (FVC). This enhancement results in an improved model, referred to as 3T_L-FVC_ model. The calculation formula for this model is as follows:

(22) $$G\lambda T=R_{n,p}\left\{(\right.1-(T_{L}\frac{1-a\left(1-FVC\right)}{FVC}\left.-T_{a})/(T_{p}-T_{a})\right\}$$

where a is the improvement factor and FVC is the fractional vegetation coverage.

**Figure 10:**
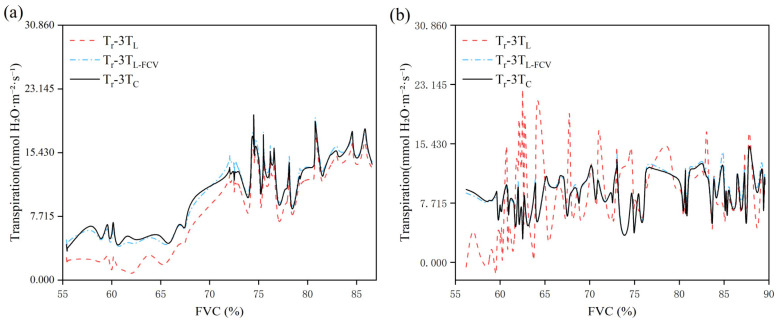
Trends of T_r_-3T_L_, T_r_-3T_L-FVC_ and T_r_-3T_C_ across different levels of FVC, (**a**) for 2022 data, (**b**) for 2023 data.

The development of trend plots for T_r_-3T_L_, T_r_-3T_C_, and T_r_-3T_L-FVC_ across different levels of FVC, as illustrated in Figure 10, indicates that variations in FVC lead to a consistent divergence of the T_r_-3T_L_ values from the T_r_-3T_C_ values. This discrepancy is likely due to the impact of soil components within T_r_-3T_L_. The improved model, T_r_-3T_L-FVC_, also presented in Figure 10, demonstrates results that are generally consistent with the T_r_-3T_C_ curve.

## 4. Discussion

### 4.1. Performance Analysis of the Improved 3T Model

Transpiration represents a significant mechanism for the exchange of water between crops, the atmosphere, and the soil [35,36]. This process is influenced by intrinsic physiological factors such as stomatal conductance, leaf area index, and crop water status, as well as by external environmental factors, including solar radiation, atmospheric conditions, and soil water status [37]. Consequently, the rapid and efficient monitoring of crop transpiration is of paramount importance for the assessing of crop water status. The 3T model has gained garnered increasing attention in both agricultural and urban studies of transpiration, primarily due to its reduced number of input parameters and relative ease of acquisition [38,39].

The results indicated that when the canopy was not full closed, the Tr estimated using the 3T_L_ model (T_r_-3T_L_) was significantly lower than estimated using the 3T_C_ model (T_r_-3T_C_). This discrepancy can be attributed to the influence of soil temperature on the LST captured by UAV-based TIR remote sensing, which affects the accuracy of the Tr estimation for the summer maize canopy.

**Figure 11:**
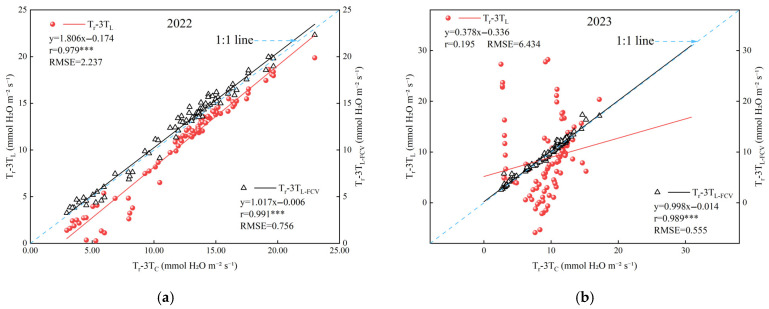
Relationship between T_r_-3T_L_, T_r_-3T_L-FVC_ and T_r_-3T_C_ (**a**) for 2022 data (n = 96), (**b**) for 2023 data (n = 132). Note: *** indicates statistical significance at *p* < 0.001.

Considering the limitations of the 3T_C_ model in practical application, this study introduced FVC into the 3T_L_ model to reduce the discrepancy between Tr-3T_L_ and Tr-3T_C_. As shown in Figure 11, the regression curves of both T_r_-3T_L-FVC_ and T_r_-3T_C_ exhibit closer alignment with the 1:1 reference line during the two-year test period, and both correlation coefficients (r) surpass those of T_r_-3T_L_ and T_r_-3T_C_. In comparison to T_r_-3T_L_ and T_r_-3T_C_, the RMSE values of T_r_-3T_L-FVC_ and T_r_-3T_C_ decreased by 66.87% and 91.38%, respectively.

These results demonstrate that the improved 3T_L-FVC_ model significantly enhances estimation accuracy and stability across annual scales. It also provides reliable transpiration estimates for large-scale farmland monitoring, offering valuable data support for water resource management and crop health monitoring.

### 4.2. Analysis of GHMRF Model Application in Thermal Infrared Images

In this study, the Gaussian Hidden Markov Random Field Model (GHMRF) was employed for the adaptive segmentation of features in TIR images of agricultural fields by utilizing both pixel greyscale information and spatial location information [40,41]. In this model, the potential energy parameter β plays a crucial role, exerting a smoothing and regulating effect on the segmented images. By encouraging neighboring pixels to adopt similar values [42], β helps improve segmentation performance in complex farmland environments, enabling more accurate extraction of canopy and soil features. As previously stated, the GHMRF model exhibits the highest segmentation accuracy at β = 0.1. Nevertheless, beyond segmentation performance, it is also essential to consider the relationship between T_C_ extracted from the TIR image and the ground-measured T_C_ [43,44].

**Figure 12:**
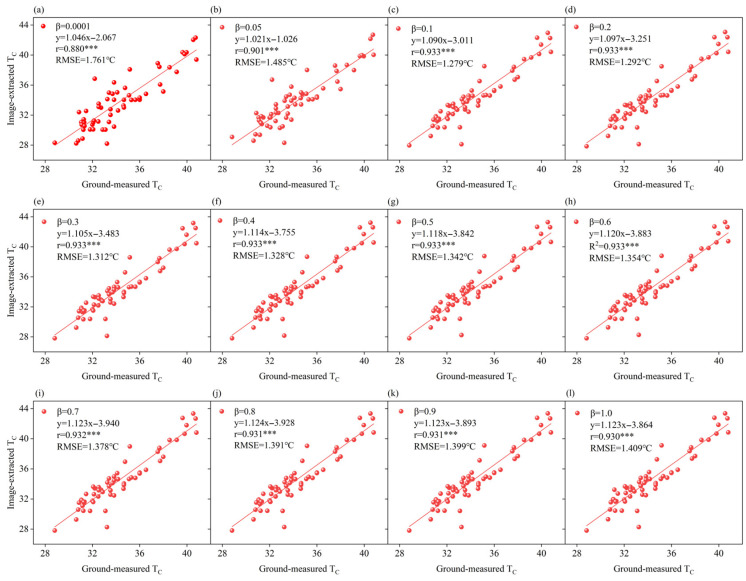
Comparative of image-extracted T_C_ and ground-measured T_C_. (**a**–**l**) Comparative of image-extracted T_C_ and ground-measured T_C_ with different β-values. Note: *** indicates statistical significance at *p* < 0.001.

To assess this, we analyzed the correlation between the extracted and measured T_C_ values (n = 60) across a range of β values. as shown in Figure 12. In all instances, a significant linear relationship was observed between the extracted and measured T_C_ values, with correlation coefficients (r) exceeding 0.88. As the β value increases, the correlation coefficient r initially rises to reach a maximum value at β = 0.1, after which it stabilized. In contrast, the RMSE initially decreases to reach a minimum value at β = 0.1 before gradually stabilized. These results demonstrate that at β = 0.1 yields the most accurate estimation of T_C_, with the highest correlation and the lowest error, consistent with the findings from the segmentation accuracy evaluation.

As the β-value increases, the model’s demand for neighboring pixel consistency also intensifies. This enhances the consistency of feature segmentation in TIR imagery; however, it concurrently leads to a loss of detail in the segmented image [45]. This phenomenon is especially pronounced in high-temperature environments and under conditions of severe stress, where the temperature of the marginal portions of the crop canopy tends to align with the temperature of the shaded soil. Consequently, this results in the isothermal mixed pixels within the TIR image [46,47]. To further investigate the influence of β-values on the GHMRF model in the context of TIR image segmentation, an analysis of the confusion matrix for segmentation results across varying β-values was conducted. The findings indicate a substantial impact of β-value variation on the segmentation frequencies of soil and canopy. Specifically, as the β-value increases, the proportion of pixels classified as soil rises, while that of canopy pixels declines. These results suggest that the GHMRF model exhibits a propensity to misclassify isothermal mixed pixels as soil with increasing β-values. Overall, the findings clarify the role of the GHMRF model in TIR image segmentation and support the selection of β = 0.1 for optimal performance in agricultural applications. This provides useful guidance for precision agriculture studies.

### 4.3. The Spatial Heterogeneity of Summer Maize T_r_

Due to the generally high spatial resolution of crop T_r_s across different soil moisture levels, these serve as essential reference points for examining crop physiological conditions and applying precision irrigation techniques [48,49,50]. However, the spatial heterogeneity of T_r_s remains an under-researched area, largely due to the coarse spatial resolution of densely planted crops [51,52].

In this study, a UAV-based approach integrating a crop mechanistic model (3T model), digital image processing (GHMRF model), and thermal infrared (TIR) imaging was developed and applied at centimeter-level resolution to assess the spatial heterogeneity of crop transpiration. It was demonstrated that there was considerable spatial heterogeneity in summer maize Tr under identical moisture treatment, particularly under the W1 moisture treatment, where the discrepancy between the maximum and minimum values could reach 6.2 mmol H_2_O m^−2^ s^−1^. This heterogeneity may be associated with the structure of the crop canopy, including factors such as leaf inclination angle and blade traits [53]. In conditions of severe water deficit, the crop exhibits a loss of turgor pressure, manifesting as leaf curling, marginal desiccation and overall wilting [54,55]. The crop center, characterized by a greater leaf inclination angle, receives reduced solar radiation and warms at a slower rate, resulting in an uneven distribution of canopy stomatal conductance. This, in turn, affects the T_C_ and transpiration of the crop. In the context of the same spatial and temporal dimensions, crop moisture status exerts a more pronounced influence on transpiration than any other factor. The spatial heterogeneity of transpiration was more pronounced under different moisture treatments, and a difference of up to 4.63 mmol H_2_O m^−2^ s^−1^ was observed between the W1 and W4 treatments. In the absence of moisture stress, the stomata of the canopy are fully open, enhancing transpiration efficiency and protecting the crop from high-temperature stress. Conversely, as moisture stress increases, the crop closes part of the stomata to reduce water consumption by transpiration, preventing excessive dehydration [56,57].

The findings suggest that a moderate water supply can facilitate crop transpiration, thereby regulating T_C_ and maintaining optimal crop growth and development. This is a crucial consideration for practical applications in farmland water management.

### 4.4. Shortcomings and Prospects

In this stud, the UAV flew at a low altitude, allowing the acquisition of high-resolution TIR images. This enabled accurate image segmentation and thus more precise estimation of FVC. However, when applying the improved 3T model over large areas, higher flight altitudes is typically required. In such cases, the accuracy of segmentation-based FVC estimation may decrease due to reduced spatial resolution. To address this, some studies have proposed estimating FVC using vegetation indices such as NDVI derived from multispectral imagery. In future research, integrating multi-source remote sensing data—including thermal, multispectral, and possibly radar sensors—could enhance the reliability and scalability of transpiration monitoring across different spatial and temporal scales.

In addition, in the 2023 growing season, only three ground-based transpiration measurements were collected, whereas twelve UAV-based observation campaigns were conducted. This limited number of ground measurements may have constrained the robustness of model validation for that year. Nevertheless, the model’s performance was still supported by accuracy assessments across different irrigation treatments. Future work should increase the frequency and number of ground-based observations to enhance validation and improve model generalizability under diverse conditions.

## 5. Conclusions

The objective of this study was to investigate the estimation of crop Tr based on UAV-based TIR remote sensing. By applying the GHMRF model for thermal image segmentation, mapping the spatial distribution of canopy temperature (TC) and Tr, and incorporating fractional vegetation cover (FVC) into the 3T model, an improved approach (T_r_-3T_L-FVC_) was developed for UAV-based monitoring. The key conclusions are:

(1) The GHMRF model is an appropriate tool for processing TIR images, and the optimal value for the potential energy parameter *β* is 0.1, which produces the most effective results for TIR image segmentation.
(2) The combination of UAV-based TIR remote sensing with a 3T model to estimate crop transpiration rates (T_r_-3T_C_) is a viable approach, although it necessitates the processing of TIR images.
(3) The introduction of FVC into the 3T model has led to the development of an improved model (T_r_-3T_L-FVC_) for estimating transpiration rates. This improved model exhibits comparable performance to the original T_r_-3T_C_ model and offers broader applicability and enhanced inter-annual stability. This novel approach provides a technological framework for the monitoring of crop T_r_s over extensive areas in a convenient, rapid, and efficient manner.

In conclusion, this study validated the viability and efficiency of UAV-based TIR remote sensing and the improved 3T model can rapidly, accurately, and efficiently estimate crop T_r_s. The approach supports large-scale, non-destructive assessments of crop water use, offering a practical tool for precision irrigation management and smart agriculture applications.

## Figures and Tables

**Table 1:** Information table of weather station monitoring sensor.

Sensor Type	Meteorological Factor	Accurate	Unit
Apogee SN500	Net radiation (R_n_)	/	W/m^2^
HC2AS3	Atmospheric temperature (T_a_) Relative humidity (RH)	±0.1 ±0.8	°C %
Lambrecht 14574	Wind speed (WS)	±1	m/s

**Table 2:** Evaluation of segmentation image accuracy for different potential energy function parameters *β*.

*β* Value	Overall Accuracy	Kaapa	User’s Accuracy	Producer’s Accuracy
0.001	88.36%	76.55%	86.32%	88.23%
0.05	91.20%	82.34%	87.60%	93.80%
0.1	94.80%	88.51%	92.06%	94.42%
0.2	94.12%	88.14%	93.30%	93.72%
0.3	93.92%	87.73%	93.20%	93.36%
0.4	93.52%	86.82%	96.99%	88.41%
0.5	93.72%	87.24%	96.56%	89.29%
0.6	92.44%	84.56%	98.16%	84.87%
0.7	92.44%	84.56%	98.16%	84.87%
0.8	92.44%	84.56%	98.16%	84.87%
0.9	92.32%	84.31%	98.15%	84.60%
1.0	92.32%	84.31%	98.15%	84.60%

**Table 3:** Descriptive distribution of transpiration rates estimated by the 3T model under different water treatments.

Water Treatments	Samples		Mean (mmol H_2_O m^−2^ s^−1^)		Minimum (mmol H_2_O m^−2^ s^−1^)		Maximum (mmol H_2_O m^−2^ s^−1^)	
	T_r_-3T_L_	T_r_-3T_C_	T_r_-3T_L_	T_r_-3T_C_	T_r_-3T_L_	T_r_-3T_C_	T_r_-3T_L_	T_r_-3T_C_
W_1-1_	40804	22193	7.737	9.966	−2.021	7.656	13.61	13.61
W_1-2_	40804	22005	7.325	9.625	−2.379	7.078	13.60	13.60
W_1-3_	40804	21777	7.768	9.991	−2.706	7.592	13.89	13.89
W_2-1_	40804	26983	9.821	11.18	−2.634	7.749	12.94	12.94
W_2-2_	40804	21593	8.233	10.44	−1.139	8.124	13.77	13.77
W_2-3_	40804	22606	8.737	10.95	−2.716	8.425	14.55	14.55
W_3-1_	40804	29362	12.35	13.13	1.836	9.702	14.65	14.65
W_3-2_	40804	25539	12.54	13.61	0.761	12.59	14.86	14.86
W_3-3_	40804	24396	10.10	11.97	−2.713	9.21	14.30	14.30
W_4-1_	40804	24792	11.87	13.37	0.116	11.74	15.42	15.42
W_4-2_	40804	30640	14.06	14.44	1.765	13.88	15.07	15.07
W_4-3_	40804	27944	13.87	14.28	4.206	13.77	14.88	14.88

## Data Availability

The data are available from the authors upon reasonable request as the data need further use.
